# Effects of hesperidin on the histological structure, oxidative stress, and apoptosis in the liver and kidney induced by NiCl_2_

**DOI:** 10.3389/fvets.2024.1424711

**Published:** 2024-06-25

**Authors:** Jinquan Chen, Xinmei Fan, Juan Chen, Xin Luo, Xin Huang, Ziling Zhou, Yue He, Shaohua Feng, Yuqing Jiao, Ruiqing Wang, Menya Ji, Jing Miao, Mengyuan Zhang, Bangyuan Wu

**Affiliations:** ^1^Department of Pharmacy, Jiangsu Food and Pharmaceutical Science College, Huai’an, China; ^2^College of Life Sciences, China West Normal University, Nanchong, China; ^3^Department of Pharmaceutical Engineering, Jiangsu Food and Pharmaceutical Science College, Huai’an, China

**Keywords:** hesperidin, nickel, liver, kidney, oxidative stress, apoptosis

## Abstract

The aim of this study was to investigate the effect of hesperidin on the liver and kidney dysfunctions induced by nickel. The mice were divided into six groups: nickel treatment with 80 mg/kg, 160 mg/kg, 320 mg/kg hesperidin groups, 0.5% CMC-Na group, nickel group, and blank control group. Histopathological techniques, biochemistry, immunohistochemistry, and the TUNEL method were used to study the changes in structure, functions, oxidative injuries, and apoptosis of the liver and kidney. The results showed that hesperidin could alleviate the weight loss and histological injuries of the liver and kidney induced by nickel, and increase the levels of lactate dehydrogenase (LDH), alanine aminotransferase (GPT), glutamic oxaloacetic transaminase (GOT) in liver and blood urea nitrogen (BUN), creatinine (Cr) and N-acetylglucosidase (NAG) in kidney. In addition, hesperidin could increase the activities of superoxide dismutase (SOD), catalase (CAT), glutathione (GSH), and glutathione peroxidase (GSH-Px) in the liver and kidney, decrease the content of malondialdehyde (MDA) and inhibit cell apoptosis. It is suggested that hesperidin could help inhibit the toxic effect of nickel on the liver and kidney.

## Introduction

Nickel is an essential element of the human body, mainly supplied by vegetables, cereals, and kelp (1). However, nickel is also a common environmental pollutant. Excessive nickel exposure can cause harm to humans. Nickel accumulates in the body through oral ingestion (food chain), respiratory inhalation, or epidermal absorption. It has been reported that nickel has obvious developmental toxicity (2) and immunotoxicity (3), which can induce nasopharyngeal carcinoma (4) and lung cancer (5). It has been reported that excessive nickel can induce lipid peroxidation in many organs (6) and intestinal microbiome disorders (7).

As the largest visceral organ in the body, the liver has a variety of complex functions, including detoxification, lipid metabolism, bile secretion, glycogen storage, and so on (8). Oxidative stress is involved in the occurrence and development of liver injury (9–11). The kidney plays the most important role in the elimination of xenobiotics (including drugs and toxic environmental factors) and is the target of heavy metals (12, 13). It has been reported that many metals can induce toxic effects on the liver or kidney, inducing oxidative stress and leading to apoptosis (14, 15). Excessive nickel exposure can cause adverse effects on the liver, including inflammatory response, oxidative damage, DNA damage, and apoptosis (16–19). The kidney is also one of the target organs of nickel injury, which plays an important role in the metabolism and excretion of nickel. Therefore, oxidative stress caused by the accumulation of nickel in the body may be one of the key mechanisms of nickel toxicity.

In recent years, the antioxidant drugs of traditional Chinese medicine have become a hot topic in different diseases or body injuries. Many studies have been carried out on the separation and identification of antioxidant components in traditional Chinese medicine, among which tangerine peel and hesperidin are essential concerns. Hesperidin (HSD) is the main component of tangerine and is a bioflavonoid mainly found in vegetables, herbs, fruits, and beans, a classic dietary polyphenol. Hesperidin is also considered to be a secondary metabolite that plays a role in plant defense against fungal and bacterial invasion (20) and has a variety of pharmacological effects, such as antioxidant (21), anti-inflammatory, anti-cancer (22), anti-diabetes, free radical scavenging, and anti-ulcer. Hesperidin can also protect the balance of intestinal microflora, improve the antioxidant and immune function of piglets, and promote their growth (23).

The toxicity of heavy metals and the accompanying health challenges are currently on the rise worldwide. Hesperidin is a natural flavonoid compound found in the genus Citrus. It has attracted wide attention due to its mild action, wide source, and no side effects. We want to understand whether hesperidin plays an important role in repairing or alleviating organ injury induced by heavy metal. Therefore, in this study, the effects of different doses of HSD on the liver and kidney of mice were investigated by intragastric administration of nickel and gradient intragastric administration of HSD. Histopathological techniques, biochemistry, and the TUNEL method were used to study the changes in structure, functions, oxidative damage, and apoptosis of the liver and kidney. We hope this study will provide a new approach to protecting and repairing liver and kidney injuries induced by excessive nickel.

## Materials and methods

### Animals and animal treatment

Kunming mice (KM) male mice (26 ± 3 g) were used in this study. They were provided by the Experimental Animal Center of North Sichuan Medical College (Sichuan, China). The mice were raised under standard conditions, temperature (23 ± 2°C) and humidity (50 ± 10%) with alternating 12 h light/dark cycles.

The animals were randomly divided into six groups, with each group containing 10 mice, shown in Table 1. The corresponding diet was attributed to them and equipped with free access to food and water by experimental requirements for 42 days. After 6 weeks, all mice were anesthetized, and blood was collected by removing the eyeballs, then euthanized by cervical dislocation. The serum was obtained by centrifugation (3,000 r/min, 15 min) to estimate the parameters related to liver and renal function. The liver and kidney tissues were collected and quickly fixed in 4% paraformaldehyde solution for histological observation and apoptosis staining. The other parts were stored at −20°C to detect antioxidant enzyme activity and the oxidative product.

**Table 1:** Animal treatment.

Groups	Treatment
Nickel + 80 mg/kg hesperidin group	Normal diet and water + Nickel and 80 mg/kg hesperidin gavage
Nickel + 160 mg/kg hesperidin group	Normal diet and water + Nickel and 160 mg/kg hesperidin gavage
Nickel + 320 mg/kg hesperidin group	Normal diet and water + Nickel and 320 mg/kg hesperidin gavage
0.5%CM Cellulose sodium salt Solution (CMC-Na, 0.5%) group	Normal diet and water + 0.5% CMC-Na
Nickel group	Normal diet and water + Nickel gavage (1.6 mg/kg 0.25 mL/day)
Control group	Normal diet and water

### Histological study in the liver and kidney

The fixed livers and kidneys were dehydrated with 50, 75, 85, 95, and 100% alcohol in order and transparentized with xylene, and wax impregnation and embedding were performed by melting paraffin. Then, sections with thicknesses of 5 μm were made. After dewaxing and dehydration, the sections were stained with hematoxylin/eosin (H.E). Finally, photographs were taken under light microscopy to observe the changes in histomorphology.

### Determination of liver function and renal function

Serum was used to assess the level of lactate dehydrogenase (LDH; A020-1-2), Alanine aminotransferase (GPT; C009-2-1), Aspartate aminotransferase (GOT; C010-1-1) in liver, creatinine (Cr; C011-2-1), blood urea nitrogen (BUN; C013-2-1) and N-acetylglucosaminidase (NAG; A031-1-1) in kidney, they were determined by the colorimetric method, Reitman–Frankel method, colorimetric method, sarcosine oxidase method, urease method, and colorimetric method. They were determined using commercially available assay kits (Nanjing Jiancheng Bioengineering Institute, Nanjing, China) according to the manufacturer’s recommended protocol.

### Assay of oxidative biochemical parameters in hepatic and renal tissue

The homogenate of the livers and kidneys was prepared and then centrifuged to obtain the supernatant. The activities of superoxide dismutase (SOD; A001-1-2), catalase (CAT; A007-1-1), glutathione (GSH; A006-1-1), glutathione peroxidase (GSH-Px; A005-1-2) and the content of malondialdehyde (MDA; A003-1-2) in the supernatant were detected according to the hydroxylamine method, ammonium molybdate method, spectrophotometric method, colorimetric method, thiobarbituric acid (TBA) method and. All the test kit purchases from Nanjing Jiancheng Bioengineering Institute (Nanjing, China) get detailed steps from instructions.

### Detection of hepatic and renal apoptosis by transferase-mediated dUTP nick-end labeling (TUNEL)

Apoptotic cells in the liver and kidney sections were detected with a colorimetric apoptosis detection kit (G001-2, TUNEL). The pretreated sample was washed twice with PBS, drying sample surrounding with absorbent paper and adding 50 μL TdT enzyme reaction solution in each sample, moisturizing the reaction in the dark at 37°C for 60 min with a cover slide, washing three times with PBS, drying the sample surrounded with absorbent paper and adding 50 μL Streptavidin-HRP working solution dropwise, moisturizing reaction in the dark at 37°C for 30 min with a cover slide, washing three times with PBS, adding 50 μL–100 μL DAB working solution, color reacting at room temperature for 10 min, washing three times with PBS. Lastly, the sections of the kidney were observed under a light microscope and got it photographed. The apoptosis cells are dark brown under a microscope.

### Statistical analysis

Data are expressed as mean ± SD of at least three independent experiments. Data were analyzed using SPSS software version 17.0. The statistical significance of the data was assessed using one-way ANOVA followed by Tukey’s test. Different lowercase letters indicate significant differences between groups (*p* < 0.05).

## Results

### Change in the weight

**Figure 1:**
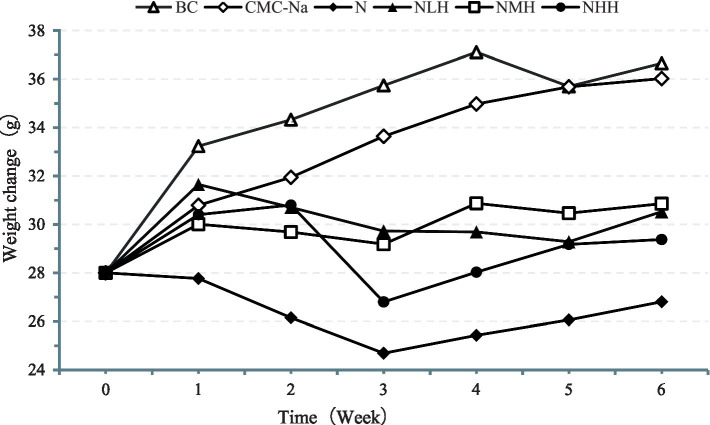
The weight of the mice. BC, Blank Control group; CMC-Na, 0.5% CM Cellulose sodium salt Solution (CMC-Na, 0.5%) group; N, Nickel group; NLH, Nickel + 80 mg/kg hesperidin group; NMH, Nickel + 160 mg/kg hesperidin group; NHH, Nickel + 320 mg/kg hesperidin group.

As shown in Figure 1, the weight of the mice was increased in the BC and CMC-Na groups but lower in the N group than in other groups, showing a trend of decreasing first and then remaining stable. The weight in the NLH group, NMH group, and NHH group showed a trend of decreasing first and then increasing.

### Histological changes in the liver and kidney

**Figure 2:**
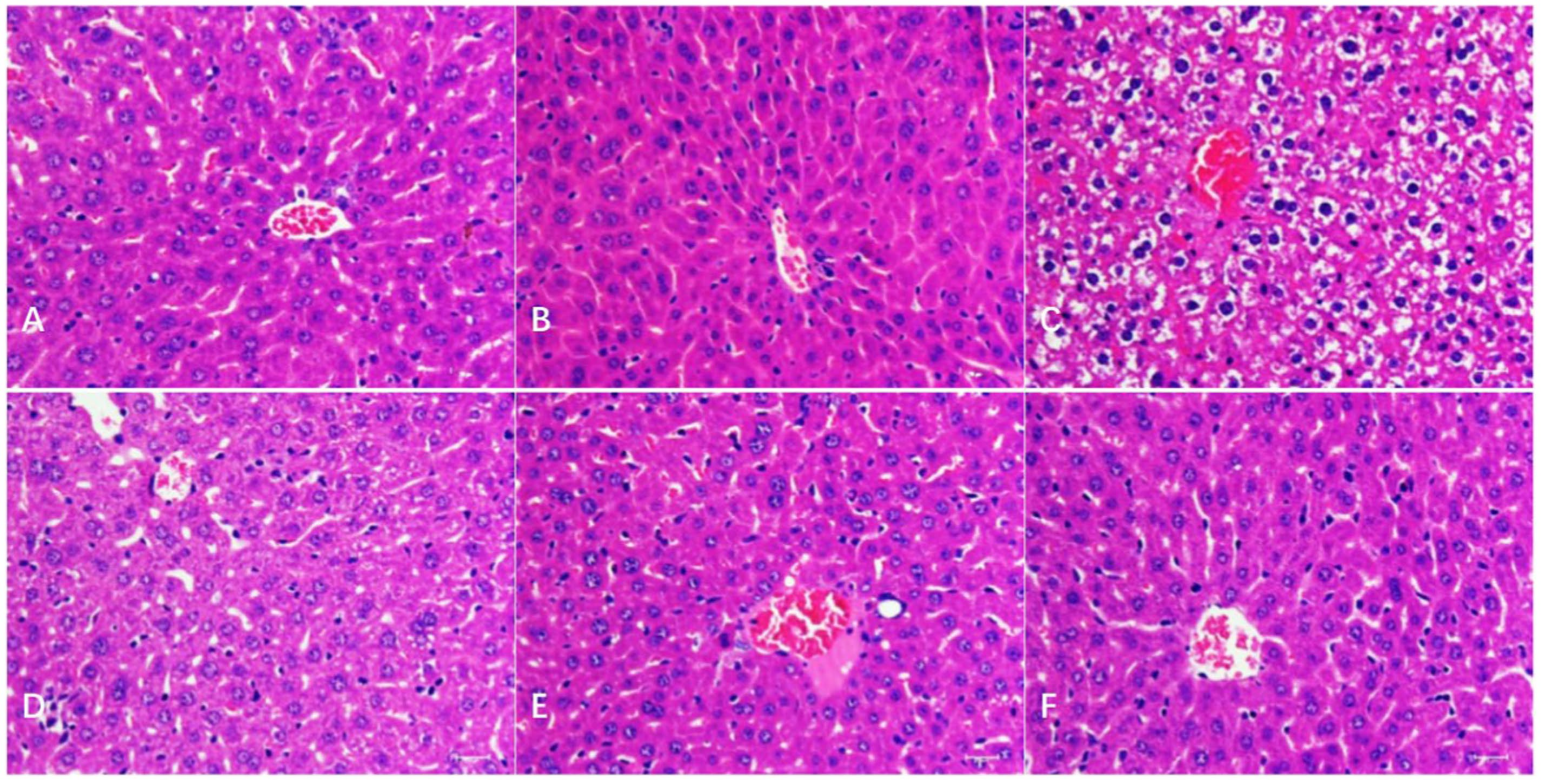
Histological changes of the liver. **(A–F)** The histological examination of H&E stained liver tissues of BC; CMC-Na; N; NLH; NMH; NHH groups (Bar = 50 μm).

As shown in Figure 2, in the liver, compared with the BC and CMC-Na, microscopically degenerative reversible lesions were observed in the N group, such as congestion and moderate vacuolar degeneration, but only slight to moderate vacuolar degeneration was observed in the NLH group. It was normal in the NMH and NHH groups compared to the BC and CMC-Na groups.

**Figure 3:**
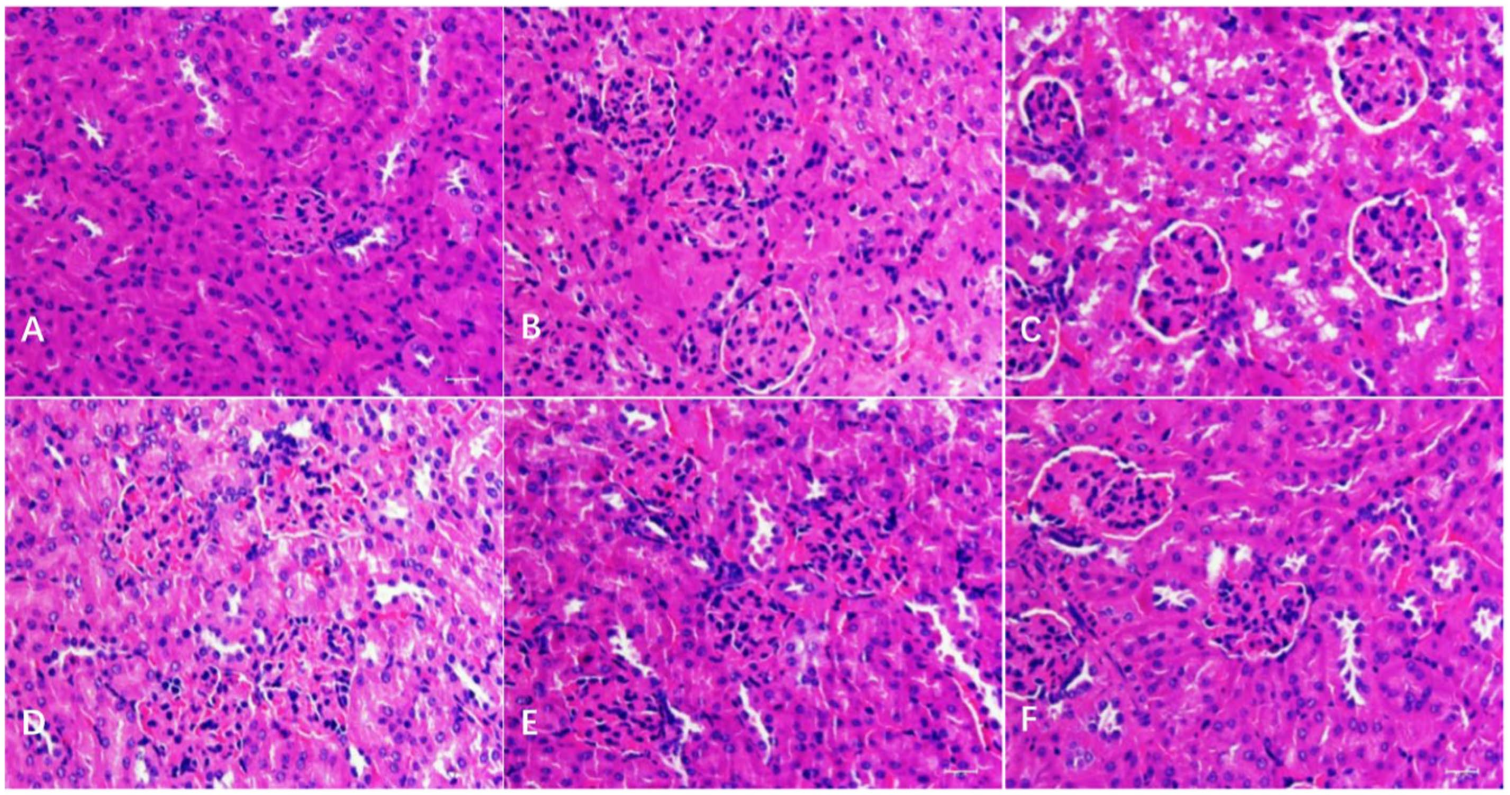
Histological changes of the kidney. **(A–F)** The histological examination of H&E stained kidney tissues of BC; CMC-Na; N; NLH; NMH; NHH groups (Bar = 50 μm).

As shown in Figure 3, in the kidney, the histopathological changes in different groups were observed. There were no obvious pathological changes in the kidney tubules and glomerulus in the BC and CMC-Na groups, and kidney tubule swelling deformation (vacuolar degeneration) was found in the N group. Compared with the N group, the injury degree in the NLH group was lower, with only slight swelling deformation, and it was normal in the NMH and NHH groups when compared with the BC or CMC-Na group.

### Changes in hepatic function and renal function

**Figure 4:**
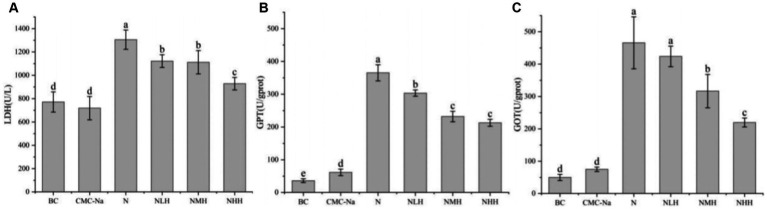
Changes in hepatic function. **(A)** LDH, lactate dehydrogenase; **(B)** GPT, glutamate pyruvate transaminase; **(C)** GOT, glutamic oxaloacetate transaminase. Different lowercase letters indicate a significant difference between groups (*p* < 0.05), while the same lowercase letters indicate no significant difference between groups (*p* > 0.05). The same as below.

The activities of LDH, GPT, and GOT were shown in Figure 4. In terms of LDH activity, there was no significant difference between the CMC-Na and BC groups and the NMH and NLH groups (*p* > 0.05). LDH activity was the highest in the N group, and LDH activity gradually decreased with the dose of hesperidin. The activity differences of GPT between each group were significant (*p* < 0.05), except for the NMH and MHH groups. The highest GPT activity was found in the N group, and the GPT activity gradually decreased with the increase in hesperidin dose (Figure 4B). The GOT activity was highest in the N group compared with BC or CMC-Na groups, and GOT activity gradually decreased with the dose of hesperidin, which can be found in Figure 4C.

**Figure 5:**
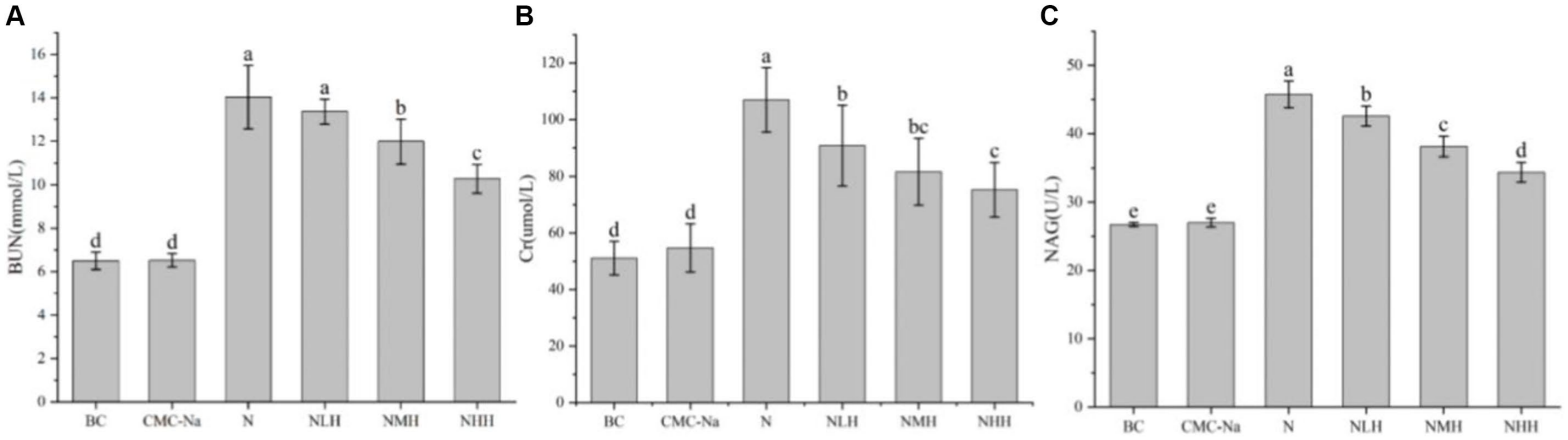
Changes in renal function. **(A)** BUN; **(B)** Cr; **(C)** NAG.

The levels of BUN, Cr, and NAG were shown in Figure 5. Compared with the BC group, the BUN level in all groups except the CMC-Na group was significantly higher (*p* < 0.05). In hesperidin groups, moderate and high concentrations of hesperidin could effectively reduce the increase of BUN level caused by nickel (*p* < 0.05)(Figure 5A). As shown in Figure 5B, the Cr level in all groups was significantly higher than that in the BC and CMC-Na groups (*p* < 0.05). Hesperidin in all dose groups can effectively reduce the increase of Cr level caused by nickel (*p* < 0.05), a significant difference between low and high dose groups (*p* < 0.05). The NAG level in all groups was significantly higher than in the BC or CMC-Na group (*p* < 0.05). Hesperidin in all dose groups could effectively reduce the increase of NAG level caused by nickel (*p* < 0.05), and there was a significant difference in different dose groups (*p* < 0.05; Figure 5C).

### Changes in oxidative stress-related parameters-antioxidant enzymes and products in the liver and kidney

**Figure 6:**
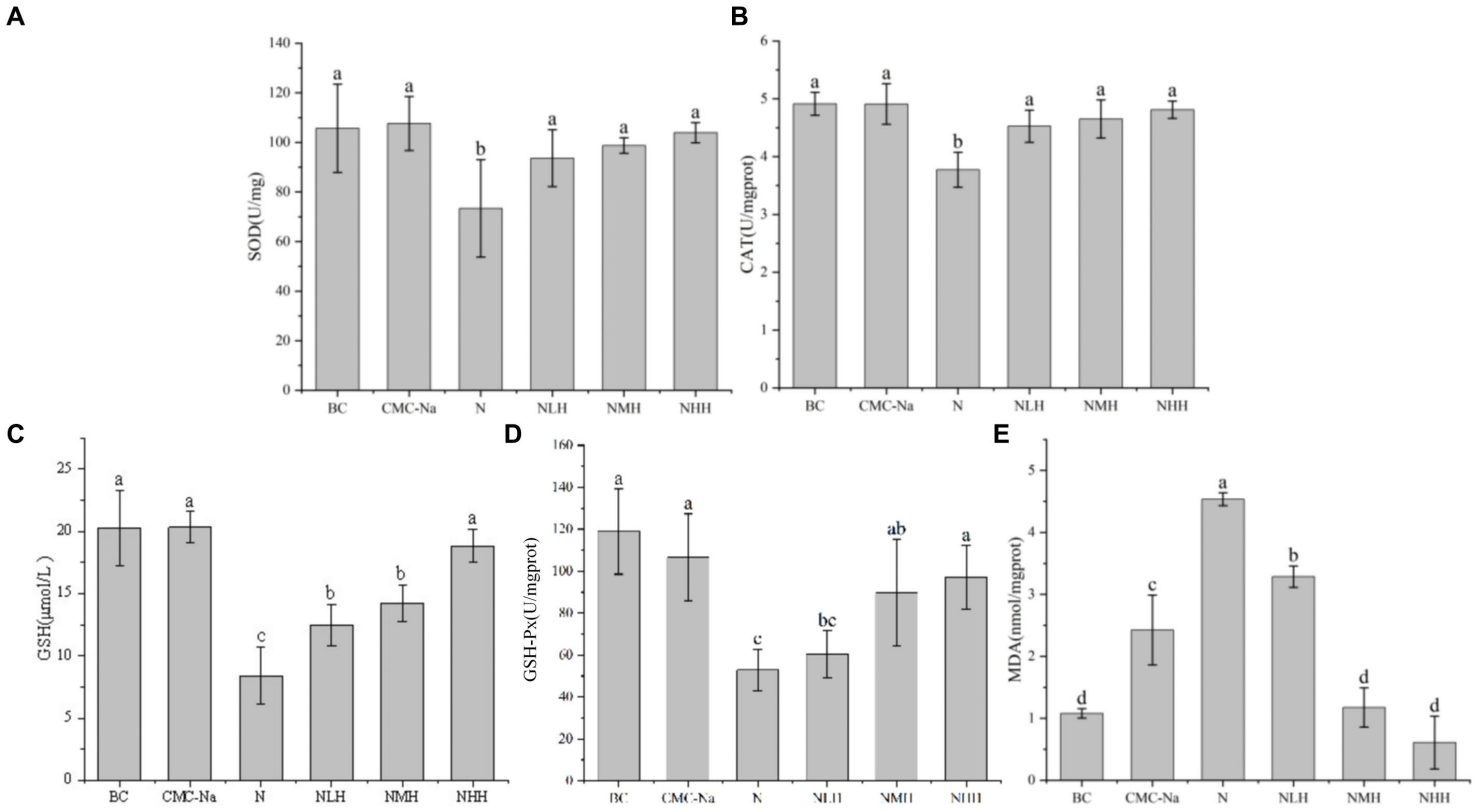
Changes in antioxidant enzymes and products in the liver. **(A)** SOD; **(B)** CAT; **(C)** GSH; **(D)** GSH-Px; **(E)** MDA.

**Figure 7:**
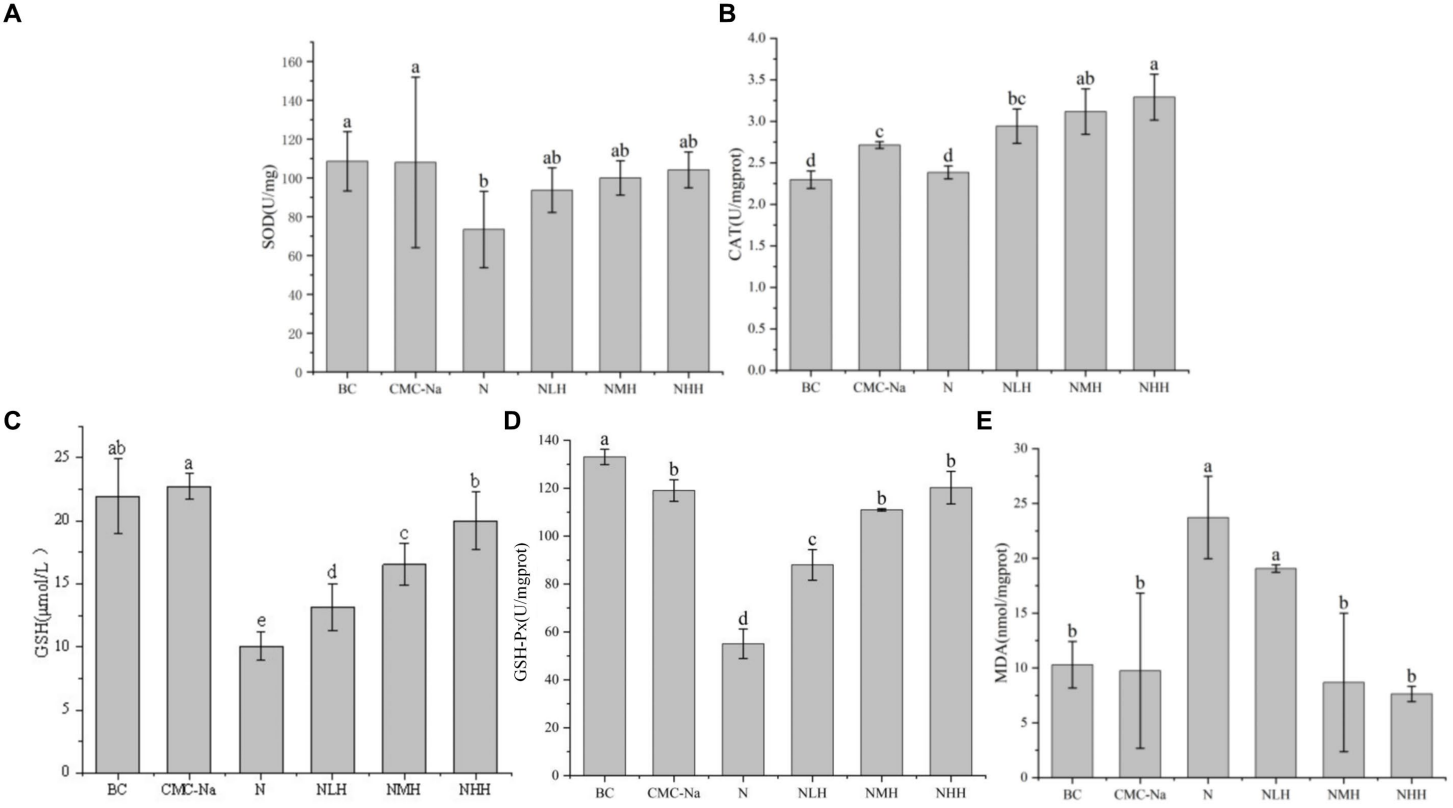
Changes in antioxidant enzymes and products in the kidney. **(A)** SOD; **(B)** CAT; **(C)** GSH; **(D)** GSH-Px; **(E)** MDA.

The peroxidation product contents and antioxidant enzyme activities were shown in Figures 6, 7. In the liver, the contents of SOD, CAT, GSH, and GSH-Px reached the lowest value in the N group, and with the increase of hesperidin dose, the activity units of each index increased, gradually tended to the BC group but always lower than the BC group. Among them, SOD reached the highest value in the CMC-Na group, GSH-Px reached the highest value in the BC group, and there were significant differences between the remaining groups (*p* > 0.05). Among the remaining indicators, there was no significant difference in CAT between the CMC-Na, NLH, NMH, and NHH groups (*p* > 0.05). There was no significant difference in GSH between the BC, CMC-Na, and NHH groups (*p* > 0.05). There was no significant difference between the NLH group and the NMH group (*p* > 0.05), and GSH content was significantly different from each other (*p* < 0.05). The difference between the CMC-Na group and other groups was statistically significant (*p* < 0.05), and the difference between the NLH group and other groups was statistically significant (*p* < 0.05), but the MDA content was always lower than that of the N group.

In the kidney, there was a statistically significant difference in SOD among the groups. The BC group was the highest value, the N group was the lowest value, and with the increase in hesperidin dose, the SOD activity unit increased, but it was always lower than the BC group. In the three different doses of hesperidin groups, the levels of GSH and CAT were highest in the NHH group, and the content of CAT and GSH increased with the increase in hesperidin dose. There were significant differences in CAT between the NHH group and BC group, CMC-Na group, N group and NLH group (*p* < 0.05), and there was no significant difference in GSH between BC group and CMC-Na group. There was no significant difference in GSH-Px among the CMC-Na, NHH, and NMH groups (*p* > 0.05). In each group, the decrease in the N group was the most obvious, and with the increase in hesperidin dose, GSH-Px activity gradually increased. The content of MDA reached the highest value in the N group. Although there was no significant difference between the N group and the NLH group (*p* > 0.05), it was significantly different from the other four groups (*p* < 0.05).

### Effects of hesperidin on Ni-induced apoptosis of liver and kidney

**Figure 8:**
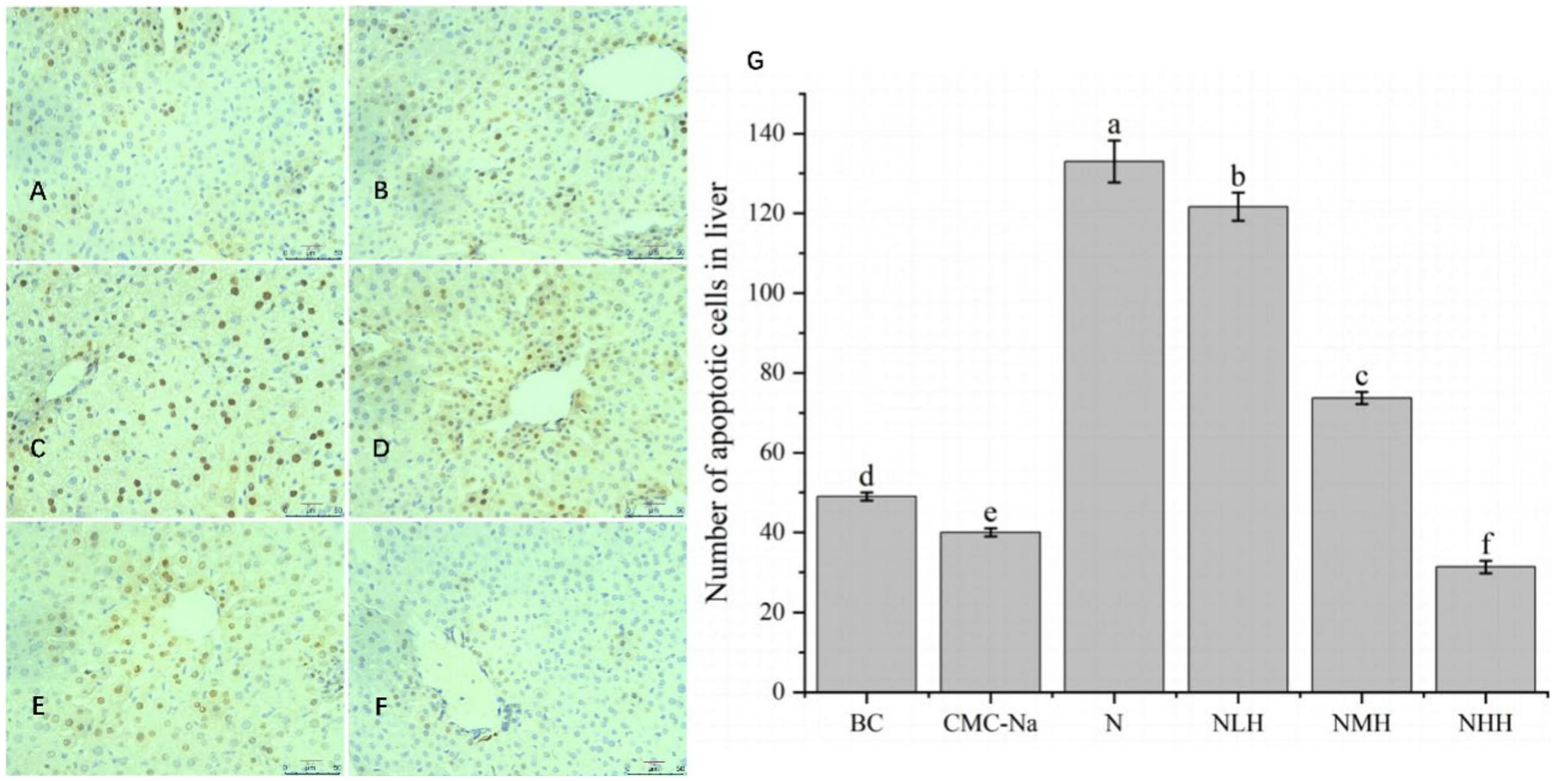
The liver is stained by TUNEL, and the apoptotic cells have brown-stain nuclei. **(A)** BC group; **(B)** CMC-Na group; **(C)** N group; **(D)** NLH group; **(E)** NMH group; **(F)** NHH group; **(G)** the number of apoptotic cells in the liver (Bar = 20 μm).

As shown in Figure 8, in the liver, the number of apoptotic cells in each group was significantly different (*p* < 0.05); compared with the BC group, the number of CMC-Na group decreased, the number of apoptotic cells in the N group was the largest, and the number of apoptotic cells in the 8NHH group was the least; compared with N group, there were significant differences between NLH, NMH and NHH groups (*p* < 0.05). The number of apoptotic cells decreased compared with the N group, and with the increase in dose, the number of apoptotic cells decreased.

**Figure 9:**
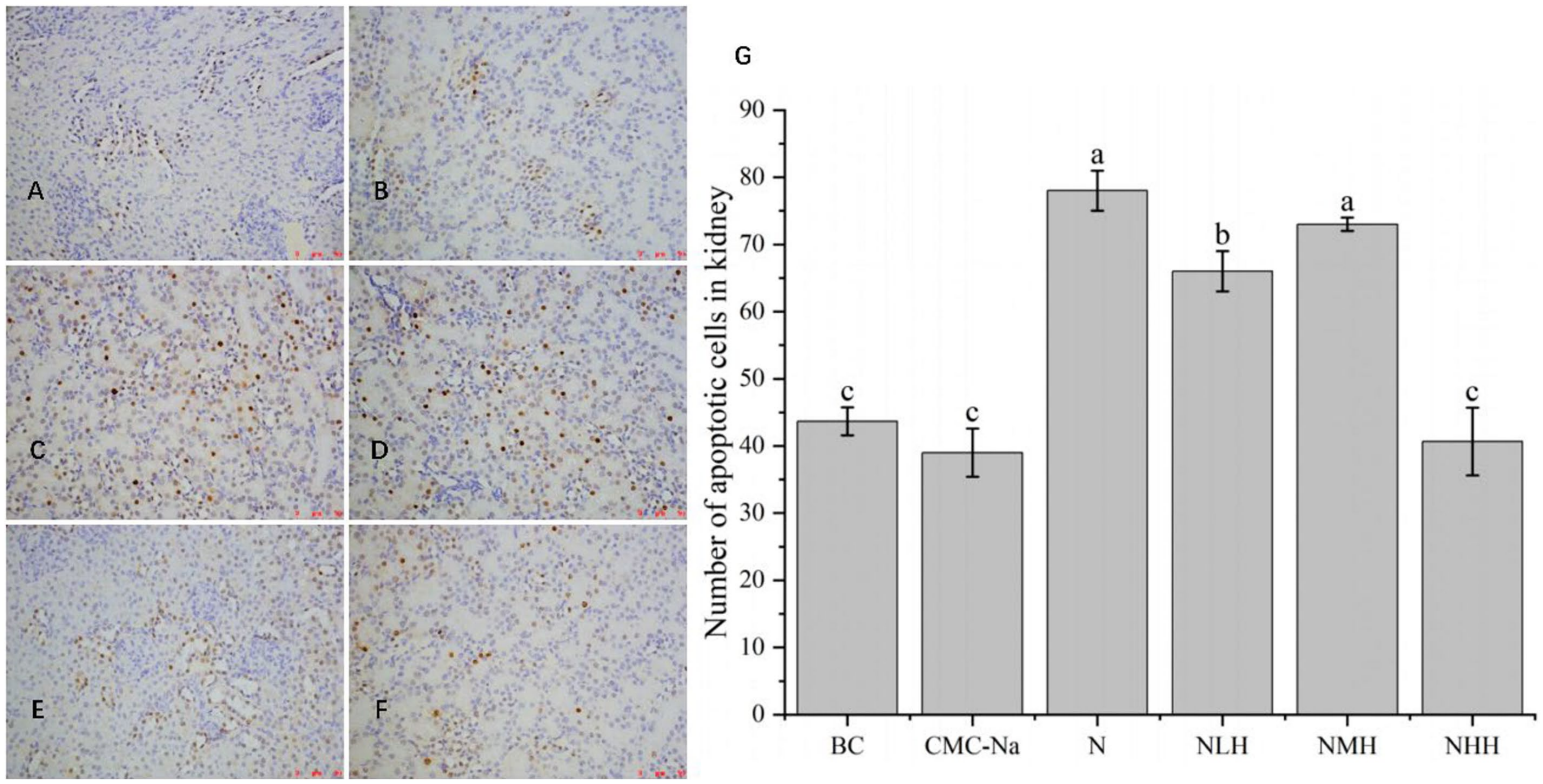
The kidney is stained by TUNEL, and the apoptotic cells have brown-stain nuclei. **(A)** BC group; **(B)** CMC-Na group; **(C)** N group; **(D)** NLH group; **(E)** NMH group; **(F)** NHH group; **(G)** the number of apoptotic cells in kidney (Bar = 20 μm).

As shown in Figure 9, in the kidney, there was no significant difference between the BC group, CMC-Na group, and NHH group (*p* > 0.05), and there was no significant difference between the N group and the NMH group (*p* > 0.05). Compared with the BC group, the number of apoptosis in the N group was the highest, and the number of apoptosis in the CMC-Na group was the lowest. The number of apoptotic cells in the NLH group, NMH group, and NHH group was lower than that in the N group and showed a trend of decreasing first, then increasing, and then decreasing, among which the number of apoptotic cells in the N group was the most.

## Discussion

Nickel, as a metal element, is found in air, water, and soil, posing potential harm to humans and animals (1). It can enter the body through the digestive tract, respiratory tract, or skin, leading to nickel accumulation in various organs. This accumulation can result in negative effects, such as hypersensitivity, inflammation, cancer, and eventually damage the body (24). Hesperidin is a natural flavonoid that exists in citrus fruits and has various biological activities, such as antioxidant, anti-inflammatory, antibacterial, or anti-cancer (25). This study aims to establish a mouse model of nickel exposure and investigate the protective effect of hesperidin on liver and kidney injury induced by nickel.

Growth and development, as one of the biological characteristics of animals, can indirectly reflect the health damage of the organism. In this study, mice were treated with nickel, and it was found that the hair gloss, activity, and appetite of the mice were decreased. With the increase of time, the body weight increased at a uniform rate from the initial stage and gradually decreased in the later stage, which indicated that nickel produced toxic effects as it accumulated in the mice. Our findings were consistent with those of nickel chloride intragastric administration after 40 days in mice (26). Following administration of hesperidin solution at doses of 80 mg/kg, 160 mg/kg, and 320 mg/kg, the body weight of mice gradually increased. Compared with the nickel treatment group, the physiological states of mice treated with hesperidin were significantly improved, and the effect was more significant with the increase of hesperidin dose.

The liver and kidney are the main organs where metal elements accumulate (27). The liver, as the important digestive gland, plays a crucial role in detoxification and metabolism, while the kidney is the main organ responsible for excretion functions (28). Therefore, the liver and kidney become the target organs of nickel toxicity and act as an essential part of body damage caused by excessive nickel. Studies have found that excess nickel compounds could cause liver and kidney damage (29, 30). In this study, mice treated with nickel chloride exhibited liver and kidney lesions, including hepatocyte granular degeneration, vacuolar degeneration, and steatosis in the liver, as well as vacuolar degeneration in the renal tubular epithelium or congested glomerular capillaries. When the mice were treated with different doses of hesperidin by gavage, the structural damage of the liver and kidney was significantly improved, indicating that hesperidin had a certain recovery effect on the liver and kidney damage caused by nickel.

The structure of tissues and organs determines their function, and the damage of the structure is bound to affect the function of organs. GPT and GOT are essential enzymes for living organisms and are mainly found in the liver. The liver is one of the major nickel-enriched organs because of its ability to produce metallothionein (MT), and MT can combine with nickel and reduce its effect on tissues (31). However, when the MT produced by the liver is insufficient to combine with Ni^2+^, excessive Ni^2+^ will cause liver damage (32). The damaged liver releases the GPT and GOT into the blood, leading to an increase in their levels (33). Therefore, the content of GPT and GOT in the blood can be used to assess the extent of liver injury. In this study, we found that the levels of GPT and GOT in the nickel treatment group were significantly increased, which showed that nickel caused injury to the liver structure and ultimately affected liver function. In addition, the levels of BUN and Cr in serum are important indicators of glomerular filtration function, which are used to diagnose kidney injury (34). The higher the levels of BUN and Cr in the serum, the more severe the damage to the kidneys. When the kidneys fail to filter BUN and Cr normally, it indicates lower renal function. In this study, the nickel treatment groups showed higher levels of GPT, GOT, BUN, and Cr in the serum compared to the control group, indicating damage to the liver and kidneys and ultimately impaired liver and kidney function. After the hesperidin treatment, the levels of these four substances in the serum are decreased. LDH is an enzyme that exists in almost all tissues and organs, including the liver and kidney (35), an important enzyme involved in glycolysis during the energy metabolism of organisms (36, 37). When the tissues and organs were damaged, the permeability of the cell membrane increases, causing more LDH to be released into the intercellular space and body fluid. Therefore, the level of LDH is one of the more sensitive indicators that reflect cell membrane damage (38). In this study, the content of LDH in the serum of nickel-treated mice was significantly increased. Combined with the results of GPT, GOT, BUN, and Cr, the changes in LDH content further reflected that the liver and kidney of mice were seriously damaged. With the addition of hesperidin in different doses, the LDH content in the serum decreased, suggesting that hesperidin had a certain restorative effect on the liver and kidney damage caused by nickel, with the highest dose of 320 mg/kg being the most effective. Although no study has directly demonstrated that hesperidin can repair the structural and functional damage to the liver and kidney, many studies have shown that hesperidin can mitigate tissue damage caused by the toxic effects of other substances, most of which are due to excessive oxidative stress, and hesperidin treatment play a protective role in this progress (39). A study found that hesperidin can protect the oxidative stress response caused by diabetic neuropathy from oxidative damage by upregulating SIRT1 and inhibiting the expression of NOX4, one of the key sources of reactive oxygen species (ROS) production (40). Therefore, we speculate that the reason why hesperidin can alleviate the injuries of the liver and kidney caused by nickel may be due to the antioxidant ability of hesperidin. Lastly, hesperidin can indirectly affect the activities of GPT, GOT, BUN, Cr, and LDH and protect the liver and kidney from the damage caused by nickel.

Nickel can affect the structure and function of the liver and kidney while also disrupting the balance between the production of ROS and the clearance of the antioxidant system. This can lead to severe oxidative damage to the body (41). ROS are produced in many aerobic cell metabolism processes. However, the concentration of ROS determines the biological effects of various intracellular targets. ROS can be significantly increased under oxidative stress. Excessive ROS is cytotoxic and mediates excessive cell apoptosis (42). Although nickel is an essential trace element for the body, excessive nickel can induce the production of ROS. Excessive ROS can cause lipid peroxidation, producing a large amount of MDA (43). MDA is a key indicator that reflects the degree of oxidative damage to the body (44). When oxidative stress occurs, ROS increases, and ROS reacts with polyunsaturated fatty acids on the cell membrane to generate lipid peroxides, eventually forming MDA (33, 45). The higher the MDA content in the liver or kidney, the more severe the oxidative damage to the tissues. Hesperidin has been shown to inhibit MDA in many studies (46, 47). The study found that compared with the control group, the content of MDA in the nickel treatment group was significantly increased, and the number of apoptotic cells was significantly increased, indicating that nickel can cause lipid peroxidation in the liver and kidney of mice, and thus the decrease of antioxidant capacity and the production of excessive ROS. The content of MDA in the liver and kidney of mice treated with hesperidin by gavage was significantly decreased, and the number of apoptotic cells decreased, indicating that hesperidin has a strong antioxidant ability and can alleviate the apoptosis caused by oxidative stress of nickel on the liver and kidney. In addition, SOD acts as the first line of defense against ROS by catalyzing the scavenging reaction of ROS through gaining and losing electrons in its metal cofactor at the enzyme’s active center, which can catalyze the dismutation reaction of superoxide (48). CAT is a ubiquitous terminal oxidase, which can catalyze the decomposition of hydrogen peroxide into oxygen and water and is a marker enzyme for peroxisomes. CAT plays an antioxidant role mainly by catalyzing the decomposition of hydrogen peroxide (49, 50). GSH-Px is an antioxidant enzyme with important biological functions, such as transmitting cell death signals by inhibiting the peroxidation of membrane phospholipids (51). GSH-Px can decompose free radical compensation products, hydrogen peroxide, and lipid peroxidation products through catalytic reduction and become non-toxic hydroxides to prevent tissue damage (52, 53). These three antioxidant enzymes play their functions and cooperate to defend against oxidative damage caused by free radicals and ROS. GSH is a kind of antioxidant in the body of organisms, mainly present in animal tissues and blood. One of its functions in cells is to resist various toxins and carcinogens with an integrated detoxification effect (42, 54). GSH plays an antioxidant role mainly by its reduced sulfhydryl group combined with free radicals in the body to convert it into acids, which are easy to metabolize and accelerate the removal of free radicals (55). In addition, GSH can also enhance the activities of SOD, CAT, GSH-Px, and other antioxidant enzymes to improve the body’s antioxidant capacity. This study showed that nickel treatment caused oxidative damage in the liver and kidney of mice, manifested as a decrease in the content of antioxidant enzymes. When hesperidin was added, the content of antioxidant enzymes in the tissues was significantly increased, and the effect was positively correlated with the dose of hesperidin. This agrees with the results of Tirkey et al. (56), who measured oxidative indices in mice after intragastric administration of different doses of hesperidin. Hesperidin belongs to the flavonoids, and they can induce the expression of protective genes by activating the Nrf2 transcriptional pathway, including coding enzymes involved in GSH synthesis (57). A study has found that hesperidin enhances the activities of antioxidant enzymes such as GR, GPT, SOD, and CAT, by affecting Nrf2 expression (58, 59). In this study, it was found that hesperidin can upregulate the activities of antioxidant enzymes such as SOD, CAT, GSH-Px, and GSH, protect cells, and reduce the severity of apoptosis. These effects are related to hesperidin’s ability to enhance Nrf2 transcription and improve antioxidant capacity.

## Conclusion

**Figure 10:**
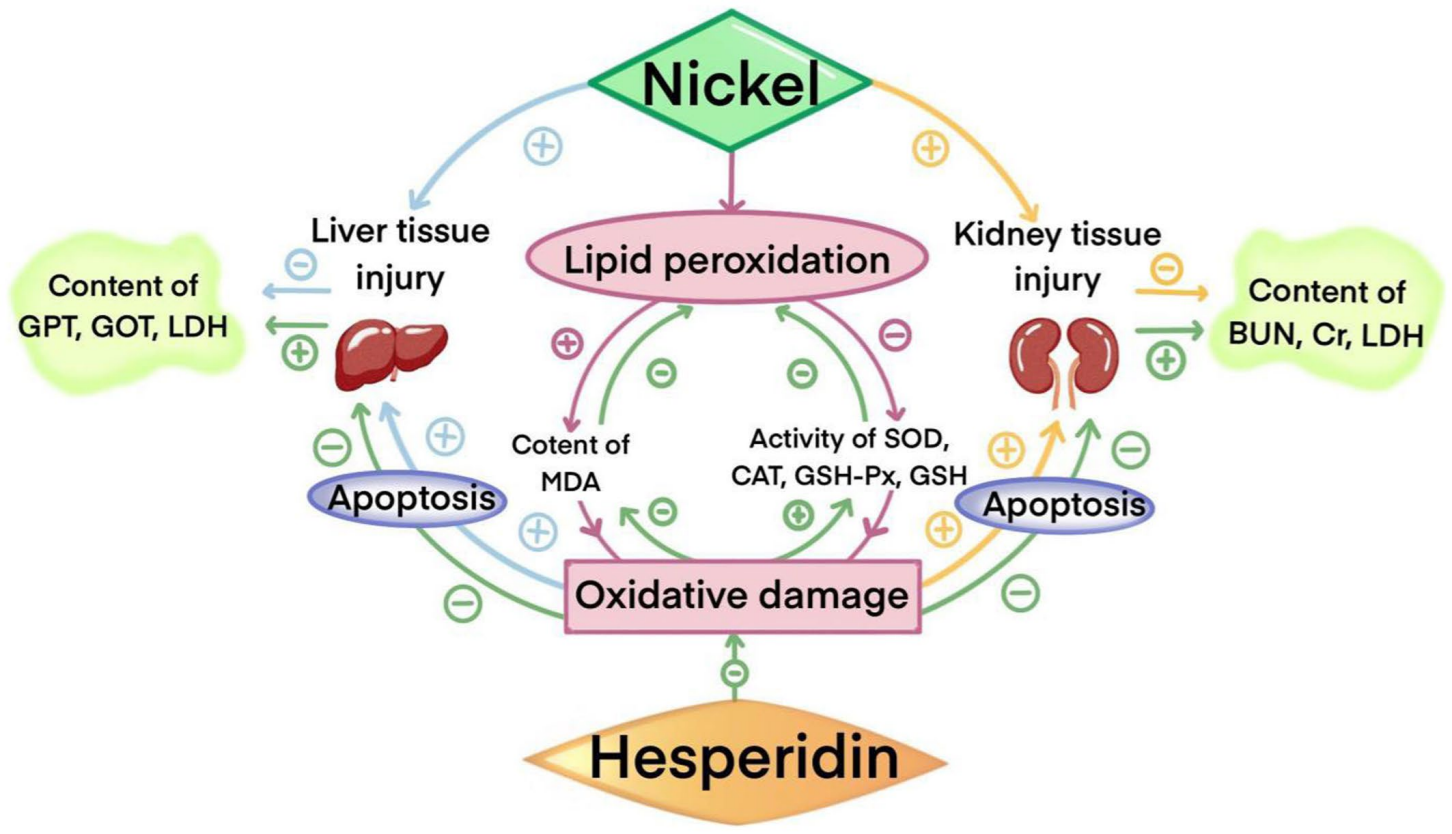
The protective effect of HSD on injuries in the liver and kidney induced by nickel.

In conclusion, hesperidin can alleviate nickel-induced liver and kidney damage, mainly through its antioxidant capacity, increasing Nrf2 transcription, effectively reducing oxidative stress and cell apoptosis, reducing the tissue damage caused by nickel, and providing beneficial protection for maintaining body health (Figure 10). Different doses of hesperidin could alleviate the damage to different degrees, and the effect was better with the increase of the dose, which provided strong experimental evidence for the potential application of hesperidin in the prevention and treatment of nickel accumulation.

## Data availability statement

The raw data supporting the conclusions of this article will be made available by the authors, without undue reservation.

## Ethics statement

The animal study was approved by the Institutional Animal Ethical Committee of China West Normal University (2024LLSC0052). The study was conducted in accordance with the local legislation and institutional requirements.

## Author contributions

JiC: Conceptualization, Formal analysis, Investigation, Methodology, Writing – original draft. XF: Data curation, Software, Writing – original draft. JuC: Methodology, Software, Writing – original draft. XL: Methodology, Software, Writing – original draft. XH: Methodology, Software, Writing – original draft. ZZ: Methodology, Software, Writing – original draft. YH: Methodology, Software, Writing – original draft. SF: Methodology, Software, Writing – original draft. YJ: Data curation, Writing – review & editing. RW: Investigation, Writing – review & editing. MJ: Validation, Writing – review & editing. JM: Validation, Writing – review & editing. MZ: Conceptualization, Funding acquisition, Project administration, Supervision, Writing – review & editing. BW: Conceptualization, Funding acquisition, Project administration, Supervision, Writing – review & editing.
